# Supplementary material for: “Physical Activity Is Not the Answer to Everything, but It Is to a Lot”: Stakeholders’ Perceived Determinants of Implementing Physical Activity Interventions for Older Adults
Source: Geriatrics (Basel). 2024 Sep 4;9(5):113. doi: 10.3390/geriatrics9050113 (PMC11417720; doi:10.3390/geriatrics9050113)
Supplement: Supplementary file 1 [file geriatrics-09-00113-s001.zip › Supplementary File S1 Interviewguide_02092024.pdf]

Interviewhandleiding Implementatie Actief Plus

In dit gesprek willen we het graag met je gaan hebben over de implementatie van sport- en beweeginterventies voor ouderen. Cijfers laten zien dat slechts een klein deel van deze groep van de bevolking voldoende beweegt. Meer bewegen staat daarom ook hoog op de prioriteitenlijst van het Nationaal Preventieakkoord. Er zijn reeds vele effectieve interventies ontwikkeld, maar het gebruik hiervan is vaak nog beperkt: de eindgebruiker wordt niet bereikt omdat de interventie door onvoldoende organisaties wordt geïmplementeerd, of niet op de juiste manier wordt geïmplementeerd. Hierbij bedoelen we met implementeren alle acties die genomen worden om er voor te zorgen dat een interventie daadwerkelijk gebruikt wordt door de doelgroep.

We willen dus in gesprek met organisaties die zelf de aangewezen partij zijn om te implementeren of met organisaties die een andere rol hebben maar goed weten wie in implementatie van dit soort interventies een rol zou moeten of kunnen spelen. Het voornaamste doel van dit gesprek is ten eerste om te achterhalen welke organisaties een rol kunnen spelen in de implementatie van sport- en beweeginterventies bij ouderen, en ten tweede wat dan die rol zou kunnen zijn. Ten derde met proberen we te achterhalen wat de overweging om een rol te spelen beïnvloed, rekening houdende met de verschillende doelen, behoeften, prioriteiten en werkprocessen van de verschillende organisaties en de invloed van de omgeving hier op. We hopen dat dit iets oplevert waar we uiteindelijk allebei iets aan hebben: wij kunnen meer kennis verzamelen over implementatietrajecten in de praktijk, die we weer kunnen verspreiden en toepassen, jullie kunnen jullie de input brengen die voor jullie van belang is, en zo ook implementatietrajecten helpen vormen op een manier die voor jullie zinvol is.

Rollen van verschillende organisaties / Netwerk

We willen graag weten welke verschillende organisaties een rol kunnen spelen in het implementeren van sport- en beweeginterventies voor ouderen en welke rol de verschillende organisaties dan kunnen spelen. Nadat we hier een goed beeld van hebben, willen we het concreter gaan hebben over de rol die jullie hierbij voor jullie organisatie zien.

1. Wanneer we het hebben over het implementeren van beweeginterventies voor ouderen, welke organisaties denk jij dan dat hierin een rol kunnen spelen? *(Hier evt werken met padlet – of zelf goed samenvatten)*
    - Denk aan lokale maar ook juist landelijke organisaties.
    - Denk hierbij ook aan verschillende rollen die organisaties kunnen hebben mbt de implementatie van een interventie: Ontwikkelen, Faciliteren, Organiseren, Informeren, Financieren. Hoe spelen zij een rol in een implementatietraject? *(Eventueel hier organisaties in de padlet in 5 clusters laten verdelen)*

- Hoe kijken jullie hierbij aan tegen commerciële organisaties? Zie je voor hun ook een rol bij de implementatie van beweeginterventies? Of wordt daar juist liever geen gebruik van gemaakt? En wat is daarvoor de reden?
- Welke van deze organisaties zijn volgens jou cruciaal om aan boord te hebben bij de implementatie van een beweeginterventie voor ouderen? Wat is de reden daarvoor?
- En welke soort persoon (functie) binnen een dergelijke organisatie dan? Wat maakt dan volgens jou dat dit de meest geschikte persoon is binnen die organisatie? Heeft deze een formele rol bij het implementeren van beweeginterventies, of is dit meer een informele rol?
- In welke mate zouden zij anderen personen of organisaties kunnen beïnvloeden?
- Er zijn nu al een heel aantal organisaties genoemd die een rol in kunnen spelen. Er zijn diverse organisaties die zich gecommitteerd hebben aan het nationaal preventie akkoord of aan het nationaal sport akkoord. (*Zorgen dat lijst van organisaties beschikbaar is*). In hoeverre denk jij dat er voor deze organisaties ook een rol is weggelegd in de implementatie van dergelijke interventies?
- Er zijn dus een heel aantal organisaties die een rol kunnen spelen in de implementatie van een beweeginterventie voor ouderen. In de praktijk kan dit betekenen dat meerdere verschillende organisaties hier tegelijk een rol in speelt. Wie of welke organisatie zou dan volgens jou de leiding hierin moeten nemen, of deze samenwerking coördineren?

2. Kun je globaal iets vertellen over met welke soort organisaties of professionals je samen werkt of informatie uitwisselt of van wie je afhankelijk bent buiten je eigen organisatie wanneer we het hebben over het implementeren van beweeginterventies, of het welbevinden van oudere doelgroepen?
    - Hoe houden jullie contact met elkaar?
    - Welke soort informatie wisselen jullie met elkaar uit?
    - In welke mate moedigt jouw organisatie je aan om te netwerken met collega's buiten je eigen organisatie?

Rollen eigen organisaties

1. Sluit het implementeren van beweeginterventies voor ouderen (on)voldoende aan bij de doelen van jullie organisatie?
    - Wat zijn deze doelen dan?
    - *Indien ouderen onderdeel zijn van hun doelen, doorvragen welke groep ouderen dit betreft:* juist de vitale ouderen, of kwetsbare ouderen?
    - Hoe komen die doelen tot stand? Komen deze bijv voort vanuit het nationaal preventie akkoord of nationaal sport akkoord? Of juist een regionaal/gemeentelijk akkoord? Of spelen nog andere factoren een rol? *(In doorvragen onderscheid maken tussen doelen intern gevormd of extern beïnvloed)*
    - Waarom sluit het wel/ niet aan bij jullie doelen?

- Hoe veranderlijk zijn deze doelen? Behoren deze bijvoorbeeld tot jullie jaarplan, en veranderen deze dus van jaar tot jaar?
- Hoe worden deze doelen gecommuniceerd en met wie?
- Hoe wordt zichtbaar of die doelen zijn bereikt? Moet dit bijvoorbeeld op een bepaalde termijn in bepaalde cijfers zijn terug te zien?
- Hoe wordt jouw organisatie afgerekend op het behalen van die doelen?
- Zit er verschil in prioriteit tussen de verschillende doelen?
  a) Hoe verhoudt het beweeggedrag van ouderen het zich tot ander prioritieten?
  b) Hoe worden die prioriteiten bepaald? Voel je een bepaalde druk om eerst andere prioriteiten aan te pakken? Waar komt dat gevoel vandaan?
  c) Hoe ga je daar mee om met de spanning die er is tussen verschillende prioriteiten?
- In welke mate denk je dat de implementatie van een beweeginterventie bij ouderen kan bijdrage aan de doelen van jouw organisatie?
- Wanneer zou je de implementatie van een interventie echt als een succes beschouwen?
- Verwacht je hierbij verschillen tussen verschillende soorten beweeginterventies voor ouderen?

2. In hoeverre vind je dat er voor jouw organisatie een rol is weggelegd bij het implementeren van beweeginterventies voor ouderen?
   - Wat is de reden dat je dat vindt?
   - En welke rol dan? (Ontwikkelen, Faciliteren, Organiseren, Informeren, Financieren)
3. We hebben het nu gehad over jullie rollen en jullie doelen. Jullie werkveld is regionaal gebonden. In hoeverre zijn er landelijk verschillen tussen vergelijkbare organisaties als die van jullie qua rollen en doelen? Worden rollen en doelen landelijk opgelegd, of hebben jullie regionaal vrijheid in het bepalen van doelen?
   - *Indien landelijk*: door gaan met volgende vragen, indien regionaal bepaald: “op basis waarvan bepalen regio’s dan hun prioriteiten?

➢ **Indien JA – men wilt zelf een rol spelen in implementatie:**

Indien NEE – ga naar pagina 5

1. In hoeverre past de implementatie van een dergelijke interventie binnen jullie huidige werkprocessen?
   - Wat zou er mogelijk moeten veranderen?
   - Of wat zou er nodig zijn om dit mogelijk te maken?
   - Hoe zou dit mogelijk gemaakt kunnen worden?
   - Verwacht je bepaalde uitdagingen?
2. In hoeverre denk je dat jouw organisatie over de beschikbare middelen bezit om een dergelijke interventie uit te zetten?
   - Voldoende tijd?

- Kennis?
- Hoe zit dit qua financiering?
- Past een dergelijke interventie binnen jullie bestaande financieringsstructuren of beschikbare subsidies?
- Wat nog meer nodig? Welke barrières verwacht je dat jouw organisatie tegen aan loopt?
- Wat zou er nodig zijn om dit passend te maken?

3. Mocht jullie organisatie een rol spelen in de implementatie van een interventie, kun je ons uitleggen hoe zo een beslissingsproces dan te werk gaan?
   - Bijv welke personen (welke functies) binnen jouw organisatie moeten hier iets van vinden?
   - Welke personen zouden verder een rol moeten spelen in de implementatie van de interventie? Bijv ondersteunend personeel?
   - Zouden ook collega's van andere afdelingen/beleidsgebieden hier mogelijk een rol in spelen? *(Bij gemeenten: Doorvragen naar mogelijke rol Ouderenbeleid / Sportbeleid / WMO-beleid)*. Beinvloed dit jouw beslissing om hier een rol in te spelen?
   - Hoe denk je dat jouw collega's daar tegen aan kijken? Hoe is de samenwerking tussen die verschillende beleidsvelden?
4. Je hebt net een aantal collega's genoemd die een rol zouden kunnen spelen binnen jouw organisatie. Kun je iets vertellen over jouw relatie met de hoofdverantwoordelijke / degene die de beslissing maakt of jullie iets met een dergelijke interventie doen?
   - In hoeverre heb jij invloed op zijn/haar ideeën? Of op de besluitvorming door die persoon?
   - Zijn er nog andere mensen binnen de organisatie die het besluit kunnen beïnvloeden.
   - Hoe verloopt zo een besluitvormingsproces in de tijd? (Bijv bepaalde formele stappen? Relevante beslissingsmomenten? Bepaalde vergadermomenten die relevant zijn?)
   - Wat is er voor nodig om een interventie vervolgens geborgd te krijgen in bijvoorbeeld het beleid?
5. Via welke kanalen binnen jouw organisatie wordt je normaal op de hoogte gebracht over nieuwe informatie, bijv nieuwe interventies, doelen die jullie bereikt hebben, nieuw personeel etc?
6. We hebben het net gehad over andere organisaties die een rol kunnen spelen in het implementeren van beweeginterventies. Beinvloed de medewerking van die andere organisaties aan de implementatie jouw beslissing om een rol te spelen in de implementatie van deze interventie?
   - Verwacht je dat andere organisaties je zullen steunen bij de implementatie van deze interventie? Op welke manier?
7. Denk je dat de implementatie van de interventie door jullie organisatie jullie voordelen op zou leveren tov andere organisaties?
8. Zijn er bepaalde lokale of nationale performance measures, en regels waaraan jullie moeten voldoen die jullie beslissing om de interventie te implementeren kan beïnvloeden? Of waarvan jullie op de hoogte zijn dat partijen die een dergelijke interventie willen implementeren mee te maken krijgen?

9. Zijn er financiële voordelen of ander soortige voordelen voor jullie die het implementeren van de interventie kan beïnvloeden?
   - Hoe kan de interventie de mogelijkheid op het verkrijgen hiervan beïnvloeden?
   - Zijn de mogelijke financiële voordelen voor jullie afhankelijk van de specifieke doelgroep waar de interventie zich op richt? Bijv verschil tussen aanpak meer vitale ouderen versus kwetsbare ouderen?
   - Heb je zicht op de financiële mogelijkheden om deze interventie te bekostigen? Bijv subsidies en vergoedingen die hier voor ingezet kunnen worden?

10. Welke indruk heb je van de drive van jullie organisatie om mee te werken vernieuwing/interventies? Kun je een voorbeeld geven?

Ga verder naar pagina 6

➢ **Indien Nee – men denkt zelf geen rol te kunnen spelen in implementatie:**

1. Je hebt eerder aangegeven dat <xxx> een cruciale rol zou kunnen spelen in de implementatie van beweeg interventies bij ouderen.
   - *Als ze het nog niet eerder genoemd hebben dan nog vragen:* Wat is de reden dat je denkt dat specifiek deze organisatie een rol kan spelen? Welke rol zouden zij moeten hebben? Is hun rol opgelegd vanuit hun takenpakket, of kunnen zij kiezen om deze rol op te pakken?
   - Welke indruk heb je van de motivatie? van deze organisatie om mee te werken aan vernieuwing/interventies? Kun je een voorbeeld geven?

2. In hoeverre denk je dat deze organisatie de beschikbare middelen bezit om een dergelijke interventie uit te zetten?
   - Voldoende tijd?
   - Kennis?
   - Hoe zit dit qua financiering?
   - Wat nog meer nodig? Welke barrières verwacht je dat deze organisatie tegen aan loopt?
   - Wat zou er nodig zijn om dit passend te maken?
3. Heb jij zich op hoe het beslissingsproces van deze organisatie om een rol te spelen in de implementatie van een interventie in zijn werk gaat?
   - Bijv welke personen (welke functies) binnen jouw organisatie moeten hier iets van vinden?
   - Welke personen zouden verder een rol moeten spelen in de implementatie van de interventie? Bijv ondersteunend personeel?
   - Zouden ook collega's van andere afdelingen/beleidsgebieden hier mogelijk een rol in spelen? Beinvloed dit jouw beslissing om hier een rol in te spelen?
   - Hoe denk je dat jouw collega's daar tegen aan kijken?
4. Heeft het implementeren van een dergelijke interventie voor deze organisatie mogelijk financiële voordelen of ander soortige voordelen die het implementeren van de interventie kan beïnvloeden?
   - Hoe kan de interventie de mogelijkheid op het verkrijgen hiervan beïnvloeden?
   - Heb je zicht op de financiële mogelijkheden om deze interventie te bekostigen? Bijv subsidies en vergoedingen die hier voor ingezet kunnen worden?
5. Zijn er bepaalde lokale of nationale performance measures en regels waarvan jullie op de hoogte zijn, waar partijen die een dergelijke interventie willen implementeren mee te maken krijgen?

➢ **Indien JA, men wilt een rol spelen in de implementatie.**
Indien NEE, ga verder naar pagina 8

Interventie behoeften

We hebben het eerder gehad over welke factoren de implementatie van de interventie mogelijk kunnen bevorderen of belemmeren. We willen hierbij nog even verder ingaan op de kenmerken van een interventie zelf, en in hoeverre deze een rol kunnen spelen bij de implementatie.

- Spelen jullie nu al een rol in de implementatie van beweeginterventies, of interventies voor ouderen in het algemeen? Wat voor soort interventies zijn dat? Waar komen deze interventies mogelijk nog te kort in?
- Door wie zijn de interventies die jullie implementeren bij voorkeur ontwikkeld? Bijv. door een praktijkorganisatie, een kennis instelling, andere organisatie?
    - Wat is de reden dat je dat vindt?
    - Hoe kijken jullie aan tegen interventies die ontwikkeld zijn op universiteiten?
    - En specifiek de OU?
    -

We willen nu graag weten wat voor jullie een interventie geschikt maakt om te implementeren. Dat zouden we graag bespreken adhv een casus, een concreet bestaande interventie, zodat wij een beter beeld kunnen vormen van wat voor jullie belangrijk is in een interventie. We maken hiervoor gebruik van de Actief Plus interventie. Dit is een interventie die ontwikkeld is door de Open Universiteit en we zouden graag van jullie horen in hoeverre dit een interventie zou zijn die zou passen binnen de verschillende doelstellingen, prioriteiten en behoeften, of waarom juist niet. En wat eventueel een interventie dan beter passend zou kunnen maken.

Actief Plus is een online of schriftelijke advies-op-maat interventie, en heeft door haar geautomatiseerde karakter de mogelijkheid om tegen zeer beperkte kosten veel mensen van een gepersonaliseerd advies te kunnen voorzien. Daarbij is deze interventie heel laagdrempelig én bewezen effectief. Aangezien de groep ouderen steeds groter wordt en beschikbare tijd steeds beperkter wordt kan dit soort interventies dus veel potentie hebben in de toekomst. Hier staat echter tegenover dat men bij deze interventie dus niet direct persoonlijk contact heeft met bijvoorbeeld een coach, en dat de interventie geen fysieke bijeenkomsten of beweegactiviteiten betreft. De verschillende voor- en nadelen van deze interventie, maar ook van andere interventies, beïnvloeden hun kans om geïmplementeerd te worden in de praktijk. Hierover wisselen we graag met jullie van gedachten.

1. Welke behoefte is er volgens jullie in de praktijk aan een dergelijke interventie?
   - Zie je die behoefte dan in het maatschappelijk belang?
   - In het belang van praktijk organisaties?
   - In het belang van jullie eigen organisatie en eigen doelen?
   - Of in het belang van de eindgebruiker?
2. We hebben het eerder gehad over verschillende organisaties die een rol kunnen spelen in de implementatie van een beweeginterventie bij ouderen. Kijk je hier anders tegen aan als het een online interventie betreft? Zie je dan nog andere relevante partijen die een rol zouden kunnen spelen?
3. De Actief Plus interventie richt zich specifiek op het bevorderen van beweeggedrag, en geeft additioneel aandacht aan het organiseren van gezamenlijke activiteiten om zo eenzaamheid bij ouderen te verminderen. In hoeverre is het van belang dat implementatie van de interventie ook kan bijdragen aan het aanpakken van andere welzijnsaspecten zoals eenzaamheid en/of zelfredzaamheid?
4. Er wordt tegenwoordig ook steeds meer gefocust op gecombineerde leefstijlinterventies: interventies waarbij zowel beweeggedrag als voedingsgedrag wordt aangepakt. In hoeverre gaat jullie voorkeur uit naar interventies waarbij meerdere gedragingen tegelijkertijd worden aangepakt?
5. In hoeverre maakt het voor jullie uit op welke laag van een ketenaanpak de interventie insteekt? (Voorkeur voor universele preventie of juist geïndiceerde preventie?) Wat is de reden hiervoor?
6. Hoe kijken jullie aan tegen online interventies voor ouderen?
7. Zoals zojuist aangegeven is de interventie bewezen effectief.
   - Hechten jullie waarde aan bewijs voor effectiviteit? Waarom is dat belangrijk? Voor wie is dat belangrijk?
   - Welk soort bewijs van effectiviteit is er nodig? In hoeverre vormt de mate van effectiviteit de doorslag om voor een bepaalde interventie te kiezen?
   - Hoe denk je dat andere stakeholders hier tegen aan kijken? (zowel binnen als buiten jouw organisatie)
8. Je hebt eerder aangegeven wanneer je de implementatie van een interventie als een succes zou beschouwen. Wat voor soort informatie zou je nodig hebben om te bepalen of dit succes bereikt is? Hoe zou je die informatie kunnen verzamelen? En op welke termijn na de implementatie zou je deze successen graag willen zien?
9. Hoe maak je een afweging hoeveel tijd en arbeid een interventie mag kosten? Hoe beïnvloed dit jullie keuze om een bepaalde interventie te implementeren?
10. Er zijn zojuist al een aantal voor- en nadelen van de Actief Plus interventie aan bod gekomen. Zie je verder nog voor- of nadelen van de Actief Plus interventie ten op zichten van andere beweeginterventies?
    - Inhoudelijk gericht op interventie kenmerken?
    - Wat als je kijkt naar de behoeftes van de doelgroep?

- En als je kijkt naar de behoeften van praktijkorganisaties?
- En organisatorisch? Denk je dat de implementatie van Actief Plus makkelijker of moeilijker is dan de implementatie van andere interventies? Wat is daarvan de reden?
- Welke barrières verwacht je tegen te komen bij de implementatie van Actief Plus?

11. Stel dat jullie een rol zouden spelen in de implementatie van Actief Plus. Op welke manier zouden jullie dan de doelgroep willen bereiken met informatie over de interventie?
    - Welke doelgroep denken jullie vooral te kunnen bereiken? De meer vitalere of juist de kwetsbare ouderen? En de digitaalvaardige, of juist de minder digitaalvaardige?
    - Zou je de interventie graag eerst aan een selecte/kleinere groep mensen aan willen bieden voordat je deze op grote schaal implementeert? Wat is daarvoor de reden?
    - Hoe zouden je plannen hiervoor uit zien?
12. Stel dat er onbeperkt geld en tijd ter beschikking is, zou je dan graag een aanpassing in deze interventie zien zodat deze beter past bij de doelstellingen van jullie organisatie en de behoeften van de praktijk?

- Indien NEE, men wilt geen rol spelen in de implementatie.

Interventie behoeften

Je hebt aangegeven dat jouw organisatie zelf geen rol kan/wilt spelen in de implementatie van beweeginterventies voor ouderen. Wij zijn benieuwd of de aard van de interventie jullie mogelijke rol in de implementatie kan beïnvloeden.

We hebben het eerder gehad over welke factoren de implementatie van de interventie mogelijk kunnen bevorderen of belemmeren. We willen hierbij nog even verder ingaan op de kenmerken van een interventie zelf, en in hoeverre deze een rol kunnen spelen bij de implementatie.

1. Wat maakt een interventie voor jullie geschikt om wel te implementeren?
2. Spelen jullie nu wel een rol in de implementatie van gezondheidsbevorderende interventies, of interventies voor ouderen in het algemeen? Wat voor soort interventies zijn dat?
3. Wanneer een interventie zich niet enkel richt op beweeggedrag, maar bijvoorbeeld ook op zelfredzaamheid, eenzaamheid of voeding, veranderd dat voor jullie de mogelijkheid om een rol te spelen in de implementatie van de interventie?
4. In hoeverre maakt het voor jullie uit op welke laag van een ketenaanpak de interventie insteekt? (Voorkeur voor universele preventie of juist geïndiceerde preventie?) Wat is de reden hiervoor?
5. Online interventies zijn makkelijker en goedkoper te implementeren, maar missen het persoonlijk contact tussen aanbieder en doelgroep. Hoe kijken jullie aan tegen online interventies in het

algemeen of specifiek voor ouderen ? Zie je daarin wel een rol voor jouw organisatie in de implementatie hier van?

Om deze laatste vraag meer concreet te maken, zouden we graag een bestaande interventie als casus aan jullie voorleggen, zodat wij een beter beeld kunnen vormen van wat voor jullie belangrijk is in een interventie. We maken hiervoor gebruik van de Actief Plus interventie. Dit is een interventie die ontwikkeld is door de Open Universiteit en we zouden graag van jullie horen in hoeverre dit een interventie zou zijn die zou passen binnen de verschillende doelstellingen, prioriteiten en behoeften, of waarom juist niet. En wat eventueel een interventie dan beter passend zou kunnen maken.

Actief Plus is een online of schriftelijke advies-op-maat interventie, en heeft door haar geautomatiseerde karakter de mogelijkheid om tegen zeer beperkte kosten veel mensen van een gepersonaliseerd advies te kunnen voorzien. Daarbij is deze interventie heel laagdrempelig én bewezen effectief. Aangezien de groep ouderen steeds groter wordt en beschikbare tijd steeds beperkter wordt kan dit soort interventies dus veel potentie hebben in de toekomst. Hier staat echter tegenover dat men bij deze interventie dus niet direct persoonlijk contact heeft met bijvoorbeeld een coach, en dat de interventie geen fysieke bijeenkomsten of beweegactiviteiten betreft. De verschillende voor- en nadelen van deze interventie, maar ook van andere interventies, beïnvloeden hun kans om geïmplementeerd te worden in de praktijk. Hierover wisselen we graag met jullie van gedachten.

13. Zoals aangegeven kost de uitvoering van een online interventie minder geld en tijd. Beinvloed dit jullie mening of jullie hier een rol in kunnen spelen?
    - Hoe maakt je een afweging hoeveel tijd en arbeid een interventie mag kosten?
14. Wat heb je al gehoord over de effectiviteit van de interventie?
    - Hechten jullie waarde aan bewijs voor effectiviteit? Waarom is dat belangrijk? Voor wie is dat belangrijk?
    - Welk soort bewijs van effectiviteit is er nodig?
    - Hoe denk je dat andere stakeholders hier tegen aan kijken? (zowel binnen als buiten jouw organisatie)
15. Zie je verder nog voor- of nadelen van de Actief Plus interventie ten opzichte van andere beweeginterventies die jullie mogelijke rol hierin kunnen beïnvloeden?
    - Inhoudelijk gericht op interventie kenmerken?
    - Wat als je kijkt naar de behoeftes van de doelgroep?
    - En als je kijkt naar de behoeften van praktijkorganisaties?
    - En organisatorisch? Denk je dat de implementatie van Actief Plus makkelijker of moeilijker is dan de implementatie van andere interventies? Waarom?
    - Welke barrières verwacht je tegen te komen bij de implementatie van Actief Plus?

16. Stel dat jullie een rol zouden spelen in de implementatie van Actief Plus. Op welke manier zouden jullie dan de doelgroep willen bereiken met informatie over de interventie?

    - Zou je de interventie graag eerst aan een selecte/kleinere groep mensen aan willen bieden voordat je deze op grote schaal implementeert? Wat is daarvoor de reden?
    - Hoe zouden je plannen hiervoor uit zien?

17. Stel dat er onbeperkt geld en tijd ter beschikking is, zou je dan graag een aanpassing in deze interventie zien zodat deze beter past bij de doelstellingen van jullie organisatie en de behoeften van de praktijk?
