# Supplementary material for: “Physical Activity Is Not the Answer to Everything, but It Is to a Lot”: Stakeholders’ Perceived Determinants of Implementing Physical Activity Interventions for Older Adults
Source: Geriatrics (Basel). 2024 Sep 4;9(5):113. doi: 10.3390/geriatrics9050113 (PMC11417720; doi:10.3390/geriatrics9050113)
Supplement: Supplementary file 1 [file geriatrics-09-00113-s001.zip › Supplementary File S2 codebook.pdf]

CODEBOEK

T.b.v. coderen interviews met stakeholders t.b.v. het implementatieproject beweeginterventies voor ouderen/Actief Plus

Onderzoeksvragen

1. Welke stakeholders/type organisaties in Brabant en Limburg zouden volgens de geïnterviewde stakeholders betrokken moeten worden/een rol moeten spelen bij het implementeren van beweeginterventies voor ouderen?
   1a Welke rol zien de geïnterviewde stakeholders in Brabant en Limburg voor zichzelf weggelegd bij het implementeren van beweeginterventies voor ouderen?
   1b Welke andere stakeholders/type organisaties zouden volgens de geïnterviewde stakeholders in Brabant en Limburg betrokken moeten worden/een rol moeten spelen bij het implementeren van beweeginterventies voor ouderen?

2. Wat zijn volgens de betrokken stakeholders belemmerende of bevorderende factoren voor de implementatie van beweeginterventies voor ouderen? Onder te verdelen in thema's op basis van de volgende CFIR domeinen:
   2a1 Welke kenmerken van beweeginterventies in het algemeen zijn volgens de stakeholders van invloed op de implementatie van beweeginterventies voor ouderen
   2a2 Welke specifieke aan Actief Plus gelinkte kenmerken zijn volgens de stakeholders van invloed op de implementatie van beweeginterventies voor ouderen
   2b Welke determinanten m.b.t hun externe setting zijn volgens de stakeholders van invloed op de implementatie van beweeginterventies voor ouderen
   2c Welke determinanten m.b.t hun interne setting zijn volgens de stakeholders van invloed op de implementatie van beweeginterventies voor ouderen
   2d Welke karakteristieken van het individu die werkzaam is bij de stakeholder zijn van invloed op de implementatie van beweeginterventies voor ouderen
   2e Welke determinanten m.b.t het implementatieproces zijn volgens de stakeholders van invloed op de implementatie van beweeginterventies voor ouderen

Enkele richtlijnen voor het coderen

- Bij elk interview zijn er een aantal vaste gegevens die gecodeerd moeten worden zoals: type organisatie en Provincie. Gebruik hiervoor de eerste coderingstabel
- **M.b.t. de codering van de CFIR determinanten**:
- **In** het codeboek zijn ter verduidelijking voorbeeldvragen uit de interviewguide opgenomen per determinant. Echter het gegeven antwoord kan ook onder een *ander* determinant vallen. Dus let bij het coderen goed op welk antwoord wordt gegeven en bij welk construct het antwoord volgens jou het beste past. Bijvoorbeeld de vraag "Is het bevorderen van het beweeggedrag volgens jou belangrijk?' staat vermeld bij de determinant 4A (individuele) kennis en attitude t.a.v. de interventie. Als de geïnterviewde uitspraken doet die betrekking hebben op de *individuele* attitude en waarde m.b.t. beweeginterventies voor ouderen, dan kun je het antwoord coderen als determinant 4A. Als de geïnterviewde in zijn antwoord ingaat op de behoefte van zijn *organisatie* aan beweeginterventies voor ouderen, dan kun je het antwoord wellicht beter coderen als determinant 3D1 (behoefte aan verandering van de organisatie).
- Het is in principe de bedoeling dat je o.a. met behulp van de gegeven inclusie- en exclusiecriteria per determinant bepaalt bij welke (*ene)* determinant het gegeven antwoord volgens jou het beste past. Je kent dus in principe *1 cod*e toe aan een antwoord. Eventueel kiezen voor dubbel coderen als je van mening bent dat 2 codes van toepassing zijn.
- Voor de determinanten 3D2 Comptabiliteit en 3D3 Relatieve prioriteit is is gekozen voor *dubbel coderen*. Uitspraken die aangeven dat een interventie wel of geen prioriteit krijgt omdat de interventie wel of niet aansluit bij de waarden en normen van de organisatie, kun je toekennen aan zowel code 3D2 als 3D3.
- Bij het coderen bij voorkeur quotes/stukken tekst selecteren bestaande uit (minimaal) 1 zin. Indien het voor de context nodig is eventueel 1 of 2 zinnen toevoegen, liever niet werken met alinea's.

1. **Welke stakeholders/type organisaties in Brabant en Limburg zouden volgens de geïnterviewde stakeholders betrokken moeten worden/een rol moeten spelen bij het implementeren van beweeginterventies voor ouderen?**

1b Welke andere stakeholders/type organisaties zouden volgens de geïnterviewde stakeholders in Brabant en Limburg betrokken moeten worden/een rol moeten spelen bij het implementeren van beweeginterventies voor ouderen?

1a Welke rol zien de geïnterviewde stakeholders in Brabant en Limburg voor zichzelf weggelegd bij het implementeren van beweeginterventies voor ouderen?

Code	Rol	Definitie	Instructie voor codering	Voorbeeld interviewvragen
R1	Ontwikkelen		-Uitspraken over de rol die de stakeholder voor zichzelf ziet weggelegd in het implementeren van een beweeginterventie voor ouderen<br>-De toelichting en argumenten die de stakeholder geeft om een bepaalde rol op te pakken bij het implementeren van een interventie	• Vind je dat er voor jouw organisatie een rol is weggelegd bij het implementeren van beweeginterventies voor ouderen?<br>• En welke rol dan? *(Ontwikkelen, Faciliteren, Organiseren, Informeren, Financieren).*<br>• En waar blijkt dat concreet uit?
R2	Faciliteren			• Idem
R3	Organiseren			• Idem
R4	Informeren			• Idem
R5	Financieren			• Idem
R6	Coördineren		-Uitspraken over de coördinatie/afstemming indien er meerdere partijen bij de implementatie van een beweeginterventie betrokken zijn<br>*-Opmerking: coördineren valt niet onder 1 van de 5 rollen (code R1 t/m R5). Onder deze code alleen*	• Wat zou er volgens u concreet moeten gebeuren m.b.t. afstemming/coördinatie als er meerdere organisaties direct betrokken zijn bij de implementatie van een

			uitspraken noteren die gaan over de coördinatie<br>*-Daarnaast altijd de rollen (code R1 t/m R5) coderen die een organisatie voor zichzelf ziet weggelegd*	beweeginterventie voor ouderen

2. **Wat zijn volgens de betrokken stakeholders belemmerende of bevorderende factoren voor de implementatie van sport- en beweeginterventies voor ouderen? Onder te verdelen in thema's op basis van de volgende CFIR domeinen:**

Domein 1 Determinanten m.b.t. kenmerken van de interventie

Code	Determinant	Definitie	Instructie voor codering	Voorbeeld interviewvragen
**1A**<br>1A1<br><br>1A2	**Innovatiebron:**<br>Beweeginterventies in het algemeen<br>Actief Plus*<br><br>**In de codering onderscheid maken tussen uitspraken die zijn gedaan over kenmerken van beweeginterventies in het algemeen en uitspraken specifiek over de kenmerken van Actief Plus*	De perceptie van key stakeholders m.b.t wie/welke organisatie de interventie heeft ontwikkeld	<u>Inclusie:</u><br>-Uitspraken over de bron van de interventie d.w.z. wie/welke organisatie de interventie heeft *ontwikkeld*<br><br><u>Exclusie:</u><br>- Uitspraken die laten zien hoe staf en deelnemers wel of niet worden *betrokken /enthousiast worden gemaakt* voor deelname aan het proces om de interventie te implementeren<br>-Bijvoorbeeld : **"**Participation in decision-making is an effective engagementstrategy to help people feel ownership of the innovation-<br>**-> <u>Engaging, betrekken en enthousiasmeren</u> 5B**	• Maakt het voor jullie iets uit door wie de interventie is ontwikkeld? Bijv. door een praktijkorganisatie, een kennisinstelling een andere organisatie/<br>• En universiteiten en specifiek de OU?
**1B**<br>1B1<br><br>1B2	**Bewijskracht en kwaliteit:**<br>Beweeginterventies in het algemeen<br>Actief Plus	De perceptie van de stakeholders m.b.t kwaliteit en validiteit van het bewijs ter ondersteuning van de overtuiging dat de interventie zal leiden tot gewenste uitkomsten	<u>Inclusie:</u><br>-Uitspraken die betrekking hebben op het bewustzijn van de sterkte en kwaliteit van het bewijs<br>-Uitspraken die betrekking hebben op het ontbreken van bewijs<br>-Uitspraken waarin een *ander*e vorm van bewijs wordt aangehaald ter ondersteuning van de overtuiging dat de interventie zal leiden tot gewenste uitkomsten , bijv.:	• Hechten jullie waarde aan bewijs voor effectiviteit? Waarom is dat belangrijk? Voor wie is dat belangrijk?<br>• Welk soort bewijs van effectiviteit is re nodig? In hoeverre vormt de mate van effectiviteit de doorslag om voor een

			-Uitspraken over resultaten vanuit een pilot/test op kleine schaal in de eigen setting i.p.v. bewijs afkomstig van wetenschappelijk onderzoek<br>-Bewijs voor effecten op een ander vlak dan op het gebied van beweeggedrag, bijvoorbeeld effecten op het gebied van positieve gezondheid, eenzaamheid, gezelligheid, groepsvorming etc.<br>- Uitspraken over ervaringen van andere organisaties met dezelfde interventie<br><br><u>Exclusie</u><br>-Uitspraken die aangeven dat het verstrekken van bewijs wordt ingezet met als doel om key stakeholders te betrekken bij de implementatie van een interventie-><br>**Engaging: Key stakeholders 5B5**<br>-Uitspraken over de (on)mogelijkheid om pilots op het gebied van implementatie uit te kunnen voeren<br>-> **Mogelijkheid om te proberen 1E**	bepaalde interventie te kiezen?<br>• Is effectiviteit het belangrijkste einddoel of gaat het om het aantal mensen dat je ermee kunt bereiken?
**1C**<br>1C1<br><br>1C2	**Relatief voordeel:**<br>Beweeginterventies in het algemeen<br>Actief Plus	De perceptie van de stakeholder van het voordeel van het implementeren van een interventie t.o.v. een alternatieve oplossing/interventie	<u>Inclusie:</u><br>-Uitspraken die aangeven dat de interventie beter of slechter is dan het bestaande programma<br>-Uitspraken over kenmerken van Actief Plus die Actief Plus wel of niet aantrekkelijker maakt om te implementeren dan andere interventies t.g.v. dat het:<br>-een advies op maat interventie is<br>-een online interventie is	• Wat zijn voor- en nadelen van de Actief Plus interventie t.o.v. andere beweeginterventies?<br>• Hoe kijk je aan dat Actief Plus een online interventie is<br>• Hoe kijken jullie aan tegen online

			-de mogelijkheid biedt om de lokale context een plek te geven in de interventie<br><br><u>Exclusie:</u><br>-Uitspraken die wijzen op de (on)mogelijkheid om de interventie aan te kunnen passen aan een bepaalde context -> **Aanpasbaarheid 1 D**<br>- Uitspraken die aangeven dat de innovatie wel of niet hoeft te worden aangepast omdat deze wel of niet aansluit bij de bestaande werkwijze en systemen -> **Comptabiliteit 3D2**<br>-Uitspraken die wijzen op een sterke behoefte aan de innovatie en/of aangeven dat de huidige situatie onhoudbaar is -> **Behoefte aan verandering 3D1**	interventies voor ouderen?<br>• In hoeverre is het voor jullie van belang dat Actief Plus ook een secundair doel heeft zoals het aanpakken van eenzaamheid en/of zelfredzaamheid?<br>• En in hoeverre heeft het de voorkeur dat Actief Plus meerdere gedragingen tegelijk aanpakt? Zoals bijv. nu wordt gedaan in de gecombineerde leefstijlinterventies waarbij zowel beweeggedrag als voedingsgedrag wordt aangepakt
**1D**<br>1D1<br><br>1D2	**Aanpasbaarheid:**<br>Beweeginterventies in het algemeen<br>Actief Plus	De mate waarin een interventie kan worden aangepast, op maat gemaakt en verfijnd kan worden om tegemoet te komen aan lokale behoeften	<u>Inclusie:</u><br>-Uitspraken die wijzen op de (on)mogelijkheid om de interventie aan te kunnen passen aan een bepaalde context<br>-Uitspraken in hoeverre het wenselijk is dat dat de interventie geïntegreerd wordt met andere activiteiten<br>-Uitspraken in de vorm van klachten over de rigiditeit van het protocol<br><br><u>Exclusie:</u>	• Stel dat er onbeperkt geld en tijd ter beschikking is, zou je dan graag een aanpassing in Actief Plus zien zodat deze beter past bij de doelstellingen van jullie organisatie en de behoeften van de praktijk?

			- Uitspraken die aangeven dat de interventie beter of slechter is dan het bestaande programma -> **Relatief voordeel 1C**<br>-Uitspraken die aangeven of een organisatie (d.w.z. de praktijkorganisatie) wel of niet een goed beeld heeft van de behoeften en middelen van de eindgebruikers van de interventie -> **Behoeften en middelen van de eindgebruikers van een interventie**<br>-Uitspraken die aangeven dat de innovatie wel of niet hoeft te worden aangepast omdat deze wel of niet aansluit bij de bestaande normen en waarden, werkwijze en systemen binnen de organisatie-> **Comptabiliteit 3D2**	
**1E**<br><br>1E1<br><br>1E2	**Mogelijkheid tot uitproberen:**<br>Beweeginterventies in het algemeen<br>Actief Plus	De mogelijkheid om de interventie op kleine schaal uit te testen in de organisatie en de mogelijkheid om de koers te veranderen c.q. de implementatie ongedaan te maken	Inclusie:<br>-Uitspraken over de (on)mogelijkheid om pilots op het gebied van implementatie uit te kunnen voeren<br><br>Exclusie:<br>- Uitspraken waarin de resultaten vanuit een pilot/test worden aangehaald ter ondersteuning van de overtuiging dat de interventie zal leiden tot gewenste uitkomsten -> **Bewijskracht en kwaliteit 1B**	• Zou je de interventie graag eerst aan een selecte/kleinere groep mensen aan willen bieden voor implementatie op grote schaal
**1F**<br>1F1<br><br>1F2	**Complexiteit:**<br>Beweeginterventies in het algemeen<br>Actief Plus	De perceptie van de mate van de moeilijkheidsgraad om de interventie te implementeren voortvloeiend uit: o.a. de duur, de scope, radicaliteit, de ingewikkeldheid en aantal te doorlopen stappen bij de implementatie	Inclusie:<br>-Uitspraken die aangeven dat het implementeren wel of niet wordt bemoeilijkt door de complexiteit van de interventie<br><br>Exclusie:<br>-Uitspraken m.b.t. beschikbaarheid van tijd en	• En organisatorisch? Denk je dat de implementatie van Actief Plus makkelijker of moeilijker is dan de implementatie van andere

			ruimte voor de implementatie-> **Beschikbare middelen 3 E2.**<br>**-**- Uitspraken die aangeven dat de interventie beter of slechter is dan het bestaande programma -> **Relatief voordeel 1C**<br>-Moeite om deelnemers te betrekken bij de implementatie-> **Betrokkenheid/Engaging: innovatiedeelnemers 5B6**	beweeginterventies voor ouderen?<br>• Welke barrières verwacht je tegen te komen bij de implementatie van Actief Plus?
**1G**<br><br>1G1<br><br>1G2	**Design kwaliteit & vormgeving :**<br>Beweeginterventies in het algemeen<br>Actief Plus	De waargenomen kwaliteit van de presentatie en vormgeving van de interventie	<u>Inclusie:</u><br>-Uitspraken m.b.t. de kwaliteit van de presentatie en vormgeving van de interventie<br><br><u>Exclusie:</u><br>-Uitspraken m.b.t de aan- of afwezigheid van de beschikbare middelen voor implementatie zoals bijvoorbeeld tijd en fysieke ruimte-> **Beschikbare middelen 3 E2.**<br>- Uitspraken die aangeven dat een goede kwaliteit en vormgeving van de interventie wordt ingezet met als doel om key stakeholders te betrekken bij de implementatie -> **Engaging/**<br>- **Betrekken en enthousiasmeren 5B**	• Geen specifieke vragen in interview guide
**1H**<br>1H1<br><br>1H2	**Kosten:**<br>Beweeginterventies in het algemeen<br>Actief Plus	Kosten van de interventie zelf en kosten om de interventie te implementeren	<u>Inclusie:</u><br>-Uitspraken m.b.t. de kosten van de interventie en de implementatie ervan<br>-<u>Exclusie:</u><br>-Uitspraken m.b.t. beschikbaarheid van tijd en ruimte voor de implementatie->**beschikbare middelen 3 E2.**	• Wat is er nodig aan randvoorwaarden om een beweeginterventie voor ouderen te implementeren in je organisatie (denk o.a. bemensing, financieel, tijd)

Domein 2: Determinanten m.b.t. externe setting

Code	Determinant	Definitie	Instructie voor codering	Voorbeeld vragen
2A	Behoeften en middelen van de eindgebruikers van een interventie	-De mate waarin een organisatie een nauwkeurig beeld heeft van de behoeften van de eindgebruikers van de interventie en de bijbehorende prioriteitstelling in die behoeften<br>-De mate waarin een organisatie een nauwkeurig beeld heeft van de barrières en facilitators bij hun eindgebruikers om in die behoeften te worden voorzien.	<u>Inclusie:</u><br>-Uitspraken die aangeven of een organisatie wel of niet een goed beeld heeft van de behoeften en middelen van de eindgebruikers van de interventie<br>-Uitspraken m.b.t. de perceptie van de behoefte aan de interventie gebaseerd op de behoeften van de eindgebruikers van de interventie en de manier waarop de interventie voldoet aan deze behoeften<br>-Uitspraken over de barrières en facilitators van de eindgebruikers m.b.t. hun deelname aan de interventie<br>-Door de eindgebruikers gegeven feedback over de tevredenheid over en succes met de interventie<br>-Uitspraken die aangeven of een nauwkeurig beeld van de behoeften van de eindgebruikers al dan niet de implementatie of adoptie van een interventie beïnvloedt<br><br><u>Exclusie:</u><br>-De mate waarin de stakeholders de huidige situatie in de eigen organisatie als intolerabel ervaren c.q. vraagt om verandering -> **Behoefte aan verandering 3D1**<br>- Uitspraken die aangeven dat de interventie/Actief Plus beter of slechter is dan het bestaande programma -> **Relatief voordeel 1C**	• Welke behoefte is er volgens jullie in de praktijk aan een interventie zoals Actief Plus?<br>• Zie je de behoefte aan Actief Plus in het belang van de eindgebruiker?<br>• Zie je verder nog voor- of nadelen van de Actief Plus interventie als je kijkt naar de behoeftes van de doelgroep? (*Afhankelijk van het gegeven antwoord coderen bij 2A of bij 1C Relatief voordeel)*

			- Uitspraken die laten zien hoe de innovatiedeelnemers wel of niet betrokken raken bij een implementatie -> **Engaging: Innovatie deelnemers5B6**	
2B	Kosmopolitisme	De mate waarin een organisatie netwerkt met andere externe organisaties	<u>Inclusie:</u><br>-Beschrijvingen van netwerkactiviteiten die plaats vinden met personen/organisaties buiten de eigen organisatie<br>-Uitspraken over de manier waarop de organisatieleden hun netwerk onderhouden en de frequentie van hun netwerkcontacten<br>-Uitspraken m.b.t de grootte van het netwerk van de organisatie en de eigen(centrale) rol en positie in dat netwerk<br>-Uitspraken over de organisaties waarmee wordt samengewerkt in het kader van het implementeren van beweeginterventies<br>-Organisaties waarnaar wordt doorverwezen t.b.v. een interview in het kader van het OU onderzoek naar beweeginterventies<br><br><u>Exclusie:</u><br>-Uitspraken over netwerken, communicatie en relaties in de *eigen* organisatie, bijv. in de vorm van meetings, email-groepen<br>-Uitspraken m.b.t.de samenstelling, de kwaliteit en het functioneren van teams *binnen* de organisatie-> **Netwerken & Communicatie 3B**	• Ten aanzien van die de organisaties die volgens jou een cruciale rol spelen in het implementeren van beweeginterventies. Hoe houd je hier contact mee? En wat voor soort informatie wisselen jullie uit<br>• Hoe belangrijk is het voor jou om een netwerk te onderhouden?<br>• Wat faciliteert dat, wat belemmert dat? (bijv. voldoende tijd)<br>• Hoe kan het best contact gehouden worden met verschillende organisaties? Met welke frequentie? Op welke manier?<br>• Welke rol speelt de medewerking van die andere organisaties binnen jullie organisatie, m.a.w. kan

				hun medewerking bepalen of jullie mee willen doen?
2C	Peer pressure	Mimetische (druk om na te bootsen) of competitieve druk om een interventie te implementeren: omdat andere collega-organisaties of concurrerende organisaties de interventie hebben geïmplementeerd of daarmee concurrentievoordeel behalen	<u>Inclusie:</u><br>-Uitspraken over waargenomen druk uitgeoefend door andere (collega) organisaties om de interventie te implementeren<br>-Uitspraken die aangeven dat een organisatie de implementatie van een interventie uitvoert op aangeven van de Gemeente /omdat de Gemeente deze interventie wil (laten) implementeren<br>-Uitspraken over waargenomen mimetische druk op basis van benchmarks<br><br><u>Exclusie:</u>	• In hoeverre is de medewerking van andere organisaties bepalend voor jullie organisatie om mee te werken aan de implementatie van beweeginterventies?
2D	Extern beleid en incentives (stimulansen/motieven)	Een breed construct dat bestaat uit verschillende factoren die de beslissing van een organisatie om een interventie te implementeren beïnvloeden waaronder:<br>-politiek beleid en regelgeving<br>-externe mandaten<br>-aanbevelingen en richtlijnen<br>-prestatiebeloning<br>-samenwerkingsverbanden<br>-publieke en benchmark rapportages	<u>Inclusie:</u><br>-Uitspraken m.b.t het implementeren van een interventie vanuit politiek oogpunt/het voorstaan op een goede politieke agenda<br>-Uitspraken over subsidies die mogelijk ingezet kunnen worden en nodig zijn om de interventie te kunnen implementeren.<br>-Uitspraken over beslissingen van de overheid (zoals bijvoorbeeld het stopzetten van de subsidie voor de MBVO-interventie) die de besluitvorming m.b.t. implementatie van een interventie beïnvloeden<br><u>Exclusie:</u>	• Hoe komen de doelen van jouw organisatie tot stand? Komen deze bijv. voort uit het nationaal preventieakkoord? Of juist een regionaal/gemeentelijk akkoord?<br>• Hoe past de implementatie van een beweeginterventie in jullie financieringsstructuur?

				• Wat is er nodig om de implementatie van de interventie te kunnen financieren bijv. bestaat er een afhankelijkheid van subsidies?<br>• Kan het implementeren van een beweeginterventie jullie ook bepaalde financiële voordelen opleveren?

Domein 3 Determinanten m.b.t. interne setting

Code	Determinant	Definitie	Instructie voor codering	Voorbeeld interviewvragen
3A	**Structurele karakteristieken**	De infrastructuur van een organisatie: sociale structuur, leeftijd, rijpheid, grootte of fysieke inrichting van de organisatie	Inclusie:<br>-Uitspraken over de infrastructuur van een organisatie en de wijze waarop het de implementatie van een interventie beïnvloedt<br>-Uitspraken over de veranderingen in de infrastructuur die nodig zijn voor de implementatie van een interventie zoals bijvoorbeeld: informatiesystemen, beslissingsprocedures en de hiërarchische structuur binnen een organisatie<br>-Uitspraken m.b.t de structuur en de stappen in het formele beslissingsproces m.b.t. de implementatie van een interventie<br>-Uitspraken m.b.t de tijdsfasering van het formele beslissingsproces: vermelding van relevante vergader- en beslismomenten<br>-Uitspraken m.b.t. de beslissingen die nodig zijn om een interventie geborgd te krijgen	• Wat is er nodig aan randvoorwaarden om een beweeginterventie voor ouderen succesvol te implementeren in je organisatie (denk aan bemensing o.a.)<br>• In welke mate is aan die randvoorwaarden gedaan ?<br>• Wat zou er nodig zijn om dit mogelijk te maken?<br>• Kun je ons vertellen hoe een formeel beslissingsproces rond de inzet van een interventie eruit ziet in je organisatie?<br>• Welke personen binnen de organisatie kunnen het besluit om te implementeren beïnvloeden c.q. zouden een rol moeten spelen in de implementatie van een interventie?<br>• Hoe verloopt een besluitvormingsproces

				in de tijd gezien: bepaalde formele stappen, relevante beslismomenten, relevante vergadermomenten?<br>• Wat is er voor nodig om een interventie geborgd te krijgen in bijvoorbeeld het beleid? Zijn hiervoor andere beslissingen nodig in het formeel besluitvormingsproces?
3B	**Netwerken & Communicatie**	De aard en kwaliteit van sociale netwerken en van de formele en informele communicatie *binnen* een organisatie	Inclusie:<br>-Uitspraken over netwerken, communicatie en relaties *in* de organisatie, bijv. in de vorm van meetings, email-groepen of andere methoden om medewerkers te verbinden of te informeren<br>-Uitspraken m.b.t.de samenstelling, de kwaliteit en het functioneren van teams *binnen* de organisatie<br>-Uitspraken over hoe de communicatie verloopt binnen het formeel beslissingsproces<br><br>Exclusie:<br>-Uitspraken m.b.t de structuur en de stappen in het formele beslissingsproces m.b.t. de implementatie van een interventie-> **3A Structurele karakteristieken**	• Als je een idee hebt om mee aan de slag te gaan, met wie bespreek je dat informeel binnen jouw organisatie<br>• Kun je ons ook vertellen hoe een formeel beslissingsproces rond de inzet van een interventie eruit ziet in je organisatie?<br>• Welke collega's binnen jouw organisatie moeten hier iets van vinden?

			- Uitspraken over de toegang tot kennis en informatie m.b.t. het gebruik van het interventie programma en de training in de achterliggende mechanismen van de interventie -> **Toegang tot kennis en informatie 3E3**<br>- Uitspraken die laten zien hoe staf en deelnemers wel of niet betrokken raken bij een implementatie en wat hun rol is m.b.t. de implementatie -> **Engaging/ betrekken en enthousiasmeren 5B**<br>-Beschrijvingen van netwerkactiviteiten die plaats vinden met personen/organisaties *buiten* de eigen organisatie<br>-> **Kosmopolitisme 2B**	
3C	**Cultuur**	**Normen en waarden en uitgangspunten van een organisatie**	Inclusie:<br>-Uitspraken over de normen en waarden en algemene uitgangspunten van een organisatie en de invloed daarvan op de implementatie van een interventie<br>- Uitspraken over de mate van interne gerichtheid (toekomstige ontwikkelingen) versus externe gerichtheid (regels en procedures m.b.t het huidig functioneren)<br>-Uitspraken over de mate van flexibiliteit (van een team, afdeling, project) versus beheersend gedrag (controle over het dagelijks werkproces)*<br><br>**Bovengenoemde factoren worden onderscheiden in het Competing Values Framework (Concurrerend Waardemodel) van Quinn en Cameron.*	• Geen specifieke vragen in interviewguide

			<u>Exclusie:</u>	
3D	**Implementatieklimaat**	**-**De mate waarin een organisatie openstaat voor verandering<br>-De mate waarin de betrokken individuele medewerkers openstaan voor de implementatie van een interventie<br>-De mate waarin het gebruik van een interventie beloond, ondersteund en verwacht wordt binnen een organisatie	<u>Inclusie:</u><br>-Algemene uitspraken over de mate waarin een organisatie openstaat voor implementatie van een interventie<br><br><u>Exclusie:</u><br>-Algemene uitspraken over de mate waarin een organisatie openstaat voor implementatie van een interventie die in te delen zijn in onderstaande **subcodes van het implementatieklimaat (3D1 tot en met 3D6)**	• Heb je collega's die veelal jouw medestanders zijn als jij een idee hebt voor een vernieuwing om mee aan de slag te gaan<br>• Heb je ook mensen om je heen van wie jij denkt dat zij hier heel anders in staan?
3D1	**Behoefte aan verandering**	De mate waarin de stakeholders de huidige situatie in de eigen organisatie als intolerabel ervaren c.q. vraagt om verandering	<u>Inclusie:</u><br>-Uitspraken die wel of niet aangeven dat er een sterke behoefte bestaat aan de innovatie in de eigen organisatie<br>-Uitspraken die aangeven dat de huidige situatie wel of niet onhoudbaar is in de eigen organisatie<br>-Uitspraken die aangeven dat de interventie wel of niet absoluut noodzakelijk is<br>-Uitspraken die aangeven dat de interventie andere programma's overbodig maakt<br><br><u>Exclusie:</u><br>- -Uitspraken die aangeven of een organisatie wel of niet een goed beeld heeft van de behoeften en middelen van de eindgebruikers van de interventie	• Is het bevorderen van het beweeggedrag volgens jou belangrijk?<br>• Denk je dat het noodzakelijk is dat er beweeginterventies worden ingezet om het beweeggedrag van ouderen te bevorderen?

			-> **Behoeften en middelen van cliënten/patiënten van een organisatie 2A**<br>-Uitspraken die aangeven dat de nieuwe interventie beter is dan bestaande interventies-> **Relatief voordeel 1C**	
3D2	**Comptabiliteit/Congruentie huidige werkwijze**	Mate waarin de interventie aansluit bij:<br>-de waarden en normen van betrokken individuen en de waarden en normen binnen de organisatie<br>- de bestaande werkwijze en systemen binnen de organisatie	Inclusie:<br>-Uitspraken over de mate van aansluiting van de interventie met de waarden en normen van betrokken individuen en de waarden en normen binnen de organisatie<br>-Uitspraken over de mate van aansluiting van de interventie bij de bestaande werkprocessen binnen de organisatie<br>- Uitspraken over de mate van aansluiting van de interventie bij de strategische doelen van de organisatie<br><br>Exclusie:<br>- Uitspraken die wijzen op de (on)mogelijkheid om de interventie aan te kunnen passen in overeenstemming met organisatiewaarden en werkprocessen -> **Aanpasbaarheid 1 D**<br><br>Dubbel coderen:<br>- Uitspraken die aangeven dat een interventie wel of geen *prioriteit* krijgt omdat de interventie wel of niet aansluit bij de waarden en normen van de organisatie -> **Relatieve prioriteit 3D3**	• Is het bevorderen van beweeggedrag van ouderen volgens jou belangrijk en wat is de reden dat jij het vindt?<br>• Stel er is onbeperkt geld en tijd ter beschikking, welke aanpassing van Actief Plus zou beter passen bij de doelstelling van jullie organisatie en behoeften van de praktijk<br>• In welke mate denk je dat het implementeren van beweeginterventies voor ouderen aansluit bij de strategische doelen van je eigen organisatie
3D3	**Relatieve prioriteit**	De gedeelde perceptie van de individuen in een organisatie	Inclusie:	• Hoe verhoudt zich het beweeggedrag van

		t.a.v. het belang van de implementatie van een interventie binnen de organisatie	-Uitspraken die de relatieve prioriteit aangeven van een interventie t.o.v. een andere interventie/andere interventies<br>-Uitspraken die de relatieve prioriteit aangeven van het gedrag dat met een interventie moet worden aangepakt, bijvoorbeeld het aanpakken beweeggedrag versus voedingsgedrag of het aanpakken van eenzaamheid<br>-Uitspraken die de relatieve prioriteit aangeven van de verschillende doelgroepen waar de interventie zich op richt, bijvoorbeeld ouderen versus jongeren<br>-Uitspraken die betrekking hebben op het veranderingsmoe zijn t.g.v. de implementatie van veel andere interventies in de organisatie<br><u>Dubbel coderen:</u><br>- Uitspraken die aangeven dat een interventie wel of geen prioriteit krijgt omdat de interventie *wel of niet aansluit bij waarden en normen van de organisatie* -><br>**<u>Comptabiliteit 3D2</u>**	ouderen zich tot andere prioriteiten in je organisatie?<br>• Heeft het bevorderen van het beweeggedrag van ouderen meer of minder prioriteit dan het beweeggedrag van andere doelgroepen?<br>• En is het wel het beweeggedrag van ouderen dat aangepakt moet worden. Of zijn er andere issues die wat jou betreft meer prioriteit hebben om aangepakt te worden binnen deze groep<br>• Hoe worden die prioriteiten bepaald?<br>• Hoe ga je daar mee om met de spanning die er is tussen verschillende prioriteiten?<br>• Verwacht je hierbij verschillen tussen verschillende soorten beweeginterventies voor ouderen?
3D4	**Organisatorische incentives /stimulansen en beloningen**	Extrinsieke incentives zoals belonen van het behalen van doelen, prestatiebeloning, promoties, salarisverhogingen	<u>Inclusie:</u><br>-Uitspraken die gaan over het bestaan van incentives of ontmoedigingsmaatregelen in relatie tot het bevorderen c.q. belemmeren	• Hoe wordt jouw organisatie afgerekend op het behalen van (strategische)doelen

			van de implementatie van een interventie c.q. de betrokkenheid van de staf bij een interventie<br><br><u>Exclusie:</u>	
3D5	**Doelen en feedback**	De mate waarin:<br>- doelen van de organisatie helder worden gecommuniceerd<br>-gehandeld wordt i.o.m. de doelen<br>-er een terugkoppeling plaatsvindt over de mate waarin doelen worden behaald<br>–doelen worden aangepast o.g.v. de gegeven feedback	<u>Inclusie:</u><br>-Uitspraken over de communicatie over de strategische doelen binnen een organisatie<br>-Uitspraken waarin feedback wordt gegeven over de mate waarin de doelen zijn behaald of niet<br>-Uitspraken die aangeven dat doelen worden aangepast o.b.v. gegeven feedback<br><br>Exclusie:<br>-Uitspraken die betrekking hebben op de reflectie over de implementatie zelf -> **Reflectie & Evaluatie 5D**	• Hoe worden de strategische doelen gecommuniceerd en met wie?<br>• Hoe wordt zichtbaar of die doelen zijn bereikt? Moet dit bijv. op een bepaalde termijn in cijfers zijn terug te zien?
3D6	**Leerklimaat**	Een klimaat waarin:<br>-de leiders zich kwetsbaar opstellen en de behoefte aan ondersteuning en input van hun teamleden aangeven<br>-teamleden zich waardevol, gewaardeerd en deskundig voelen m.b.t het veranderingsproces<br>-Individuen voelen zich veilig om nieuwe zaken uit te proberen<br>-Er is genoeg tijd en ruimte voor reflectie en evaluatie	<u>Inclusie:</u><br>-Uitspraken die wel of niet betrekking hebben op de mate waarin binnen een organisatie sprake is van een leerklimaat (zie definitie)<br><br><u>Exclusie:</u>	• Geen specifieke vragen in interviewguide

3E	**Mate van bereidheid tot implementatie**	Concrete en directe aanwijzingen voor de aanwezigheid van commitment binnen de organisatie m.b.t. de beslissing om de interventie te implementeren binnen de organisatie	Inclusie:<br>-Uitspraken die wijzen op de bereidheid van de organisatie tot implementatie<br><br>Exclusie:<br>-Uitspraken die wijzen op de bereidheid tot implementatie en die in te delen zijn in de **subcodes 3E1, 3E2, 3E3**	• Vind je dat er voor jouw organisatie een rol weggelegd is bij het implementeren van beweeginterventies voor ouderen?<br>• En welke rol dan?
3E1	**Leiderschap engagement**	Commitment, betrokkenheid en verantwoordelijkheidsgevoel van (reguliere) leidinggevenden van de organisatie m.b.t. de implementatie van de interventie	Inclusie:<br>-Uitspraken die de mate van betrokkenheid van (reguliere) leidinggevenden binnen een organisatie aangeven m.b.t. de implementatie van de interventie<br><br>Exclusie:<br>-Uitsluiten uitspraken die betrekking hebben op leidinggevenden die leiding geven aan het implementatietraject -> **Betrokkenheid/Engaging 5B: Formeel aangestelde implementatie leiders 5B2: Champions 5B3**	• Geen specifieke vragen in interviewguide
3E2	**Beschikbare middelen**	De beschikbare middelen bestemd voor implementatie en doorlopende bedrijfsvoering inclusief fysieke ruimte en tijd	Inclusie:<br>-Uitspraken m.b.t. de aan-en afwezigheid van beschikbare middelen specifiek bestemd voor de implementatie van de interventie<br><br>Exclusie:<br>-Uitspraken die gaan over training en educatie -> **Toegang tot kennis en informatie 3E3**<br>-Uitspraken over de kwaliteit van materialen -> **Design kwaliteit & vormgeving 1G**	• Wat is er nodig aan randvoorwaarden om een beweeginterventie voor ouderen succesvol te implementeren in je organisatie (denk aan bemensing, financieel, tijd, prioriteit)<br>• Wat zou er mogelijk moeten veranderen?<br>• Wat is er voor nodig om een interventie

				geborgd te krijgen in bijvoorbeeld het beleid mbt beschikbare middelen?<br>• Wat zou er nodig zijn om dit mogelijk te maken<br>• Verwacht je bepaalde uitdagingen? Bijv. omdat de processen botsen met de beschikbare middelen (tijd, kennis)
3E3	**Toegang tot kennis & Informatie**	De toegankelijkheid van bruikbare informatie en kennis over de interventie en hoe de interventie inpasbaar is in de werktaken	**Inclusie:**<br>**-**Uitspraken over de toegang voor implementatieleiders en gebruikers tot kennis en informatie m.b.t. het gebruik van het interventie programma en de training in de achterliggende mechanismen van de interventie<br><br>Exclusie:<br>-Uitspraken over hoe key stakeholders betrokken worden bij de interventie en wat hun rol is bij het implementeren van de interventie **-> Engaging; Key stakeholders 5B5**<br>- Uitspraken over netwerken, communicatie en relaties *in* de organisatie, bijv. in de vorm van meetings, email-groepen of andere methoden om medewerkers te verbinden of te informeren -> **Netwerken & Communicatie 3B**	• Geen specifieke vragen in interviewguide

			-Uitspraken m.b.t.de samenstelling, de kwaliteit en het functioneren van teams binnen de organisatie -> **Netwerken & Communicatie 3B**	

Domein 4 determinanten m.b.t. karakteristieken van het individu

Code	Determinant	Definitie	Instructie voor codering	Voorbeeld vragen
4A	**Kennis en attitude t.a.v. de interventie**	-Iemands individuele attitude t.o.v. en de waarde die wordt gehecht aan een interventie<br>-Iemands bekendheid met feiten, waarheden en principes m.b.t. de interventie	<u>Inclusie:</u><br>**-** Uitspraken die betrekking hebben op de individuele attitude en waarde m.b.t. beweeginterventies voor ouderen<br><br><u>Exclusie:</u><br>Uitspraken over de bekendheid met de bewijsvoering voor de interventie-> **1B Bewijskracht en kwaliteit**	• Is het bevorderen van het beweeggedrag volgens jou belangrijk?<br>• En wat is de reden dat jij dat vindt<br>• En hoe belangrijk vind je dat dan?
4B	**Eigen-effectiviteit**	Mate waarin het individu zich in staat acht om de acties die nodig zijn om de implementatie doelen te bereiken uit te voeren	<u>Inclusie:</u><br><br><u>Exclusie:</u>	• Welke barrières verwacht je tegen te komen bij de implementatie van Actief Plus?
4C	**Individuele stadium van verandering**	-Mate waarin het individu vorderingen maakt in het deskundig, enthousiast en duurzaam gebruik van de interventie<br>- Uitgedrukt in de indeling van het individu in de fasen van verandering (van Prochaska):<br>1. Voorbeschouwing	<u>Inclusie:</u><br><br><u>Exclusie:</u>	• Geen specifieke vragen in interviewguide

		2. Overpeinzing<br>3. Voorbereiding<br>4. Actieve verandering<br>5. Stabilisatie		
4D	**Individuele identificatie met de organisatie**	Een breed concept dat aangeeft hoe individuen de (relatie met de) organisatie ervaren en de mate van commitment met de organisatie	Inclusie<br><br>Exclusie	• Geen specifieke vragen in interviewguide
4E	**Overige persoonlijke attributies**	Nader in te vullen?		

Domein 5 Determinanten m.b.t het implementatieproces

Code	Determinant	Definitie	Instructie voor codering	Voorbeeld vragen
5A	**Planning**	De mate waarin van te voren een planning is gemaakt van de taken en acties die moeten worden doorlopen t.b.v. het implementeren van een interventie	Inclusie:<br>-Uitspraken die laten zien dat voor de implementatie een diagnostisch onderzoek is uitgevoerd en een planning is gemaakt<br>-Uitspraken m.b.t. het aanbrengen van verbeteringen in de gemaakte planning<br><br>Exclusie:	• Geen specifieke vragen in interviewguide
5B	**Engaging/ betrekken en enthousiasmeren**	-Het aantrekken en het boeien van geschikte individuen voor de uitvoering en implementatie van de interventie<br>-D.m.v. toepassing van een gecombineerde strategie bestaande uit o.a. sociale marketing, educatie, rolmodellen	Inclusie:<br>-Uitspraken die laten zien hoe staf en deelnemers wel of niet worden betrokken /enthousiast worden gemaakt voor het implementeren van een interventie en de rol die zij daarin spelen<br>-Bijvoorbeeld : **“**Participation in decision-making is an effective engagementstrategy to help people feel ownership of the innovation**”**<br><br>Exclusie:<br>-Uitspraken die vallen onder een specifieke subcategorie**, bijv. Champions 5B3 of opinieleiders 5B1**<br>**-** Uitspraken over de bron van de interventie d.w.z. wie/welke organisatie de	• Geen specifieke vragen in interviewguide

			interventie heeft ontwikkeld-> **Innovatie bron 1A**	
**5B1**	**Opinieleiders**	Individuen in een organisatie die een formele of een informele invloed hebben op de attitudes en overtuigingen van hun collega's t.a.v. de implementatie van een interventie	Inclusie:<br>- Uitspraken die laten zien hoe de opinieleiders wel of niet betrokken raken bij een implementatie en wat hun rol is m.b.t. de implementatie	• Als jij een idee hebt om mee aan de slag te gaan, met wie bespreek je dit dan informeel binnen jouw organisatie? Heb je collega's die veelal jouw medestanders zijn op dit gebied?<br>• Kun je ons ook vertellen hoe een formeel beslissingsproces rond inzet van een interventie eruit ziet in je organisatie? Welke personen/functies binnen jouw organisatie moeten hier iets van vinden?
**5B2**	**Formeel aangestelde interne implementatie leiders**	-Individuen die formeel aangesteld zijn en verantwoordelijkheid dragen voor de implementatie:<br>-In de rol van coördinator, projectmanager, teamleider of in andere vergelijkbare rollen	Inclusie:<br>-Uitspraken die laten zien hoe de formeel aangestelde implementatieleiders wel of niet betrokken raken bij een implementatie en wat hun rol is m.b.t. de implementatie<br>**-**Exclusie:<br>Uitspraken die de mate van betrokkenheid aangeven van leidinggevenden binnen een organisatie die geen taak hebben als implementatieleider -> **Leadership Engagement** 3 E1	• Welke personen zouden een rol moeten spelen in de implementatie van de interventie?<br>• Ondersteunend personeel? Collega's van andere beleidsgebieden/afdelingen?
**5B3**	**Champions**	Individuen die zich inzetten voor het steunen, het vermarkten en het tot een goed einde brengen/het	Inclusie:<br>Uitspraken die laten zien hoe de champion wel of niet betrokken raakt bij een	• Geen specifieke vragen in interviewguide

		afronden van de implementatie ongeacht de weerstand en onverschilligheid die een interventie kan oproepen in een organisatie	implementatie en wat haar/zijn rol is m.b.t. de implementatie<br><br><u>Exclusie:</u><br>Uitspraken die aangeven dat een champion eveneens een (reguliere) leidinggevende functie heeft in de organisatie -> **Leadership Engagement** 3 E1	
**5B4**	**Externe change agents**	Individuen die zijn verbonden aan een externe organisatie en die de beslissingen m.b.t. de interventie formeel beïnvloeden of faciliteren in de gewenste richting	<u>Inclusie:</u><br>-Uitspraken die laten zien hoe de externe change agents wel of niet betrokken raken bij een implementatie en wat hun rol is m.b.t. de implementatie c.q. hoe zij implementatie inspanningen ondersteunen<br><br><u>-Exclusie:</u><br>-Uitspraken m.b.t. ondersteunende activiteiten zoals het geven van een training in de mechanismen van het programma waarbij de training wordt gegeven door een interne medewerker **-> Toegang tot kennis & informatie 3E3**	• Welke andere organisaties zouden volgens jou een cruciale rol moeten spelen in het aanbieden van beweeginterventies voor ouderen<br>• Welke rol speelt de medewerking van andere organisaties binnen jullie organisatie, m.a.w. kan hun medewerking bepalen of jullie mee willen doen?
**5B5**	**Key stakeholders**	Individuen vanuit de interne organisatie die direct worden beïnvloed door de interventie c.q. de staf die verantwoordelijk is voor het doen van aanbevelingen oor de nieuwe interventie	<u>Inclusie:</u><br>-Uitspraken die laten zien hoe de key stakeholders die belangrijk zijn voor de implementatie van een interventie wel of niet betrokken raken bij een implementatie en wat hun rol is m.b.t. de implementatie<br><br><u>Exclusie:</u><br>-Uitspraken over de toegang tot kennis en informatie m.b.t. het gebruik van het	• Welke personen zouden een rol moeten spelen in de implementatie van de interventie?<br>• Ondersteunend personeel? Collega's van andere beleidsgebieden/afdelingen

			programma, inclusief een training in de mechanismen van het programma -> **Toegang tot kennis & informatie 3E3**<br>-_Uitspraken over netwerken in het algemeen, communicatie en relaties in de organisatie , bijv. in de vorm van meetings, email-groepen of andere methoden om medewerkers te verbinden of te informeren<br>-Uitspraken m.b.t.de samenstelling, de kwaliteit en het functioneren van teams binnen de organisatie -> **Netwerken en communicatie -> 3B**	
**5B6**	**Innovatie deelnemers**	De stakeholders en eindgebruikers van een interventie	<u>Inclusie:</u><br>-Uitspraken die laten zien hoe de stakeholders en eindgebruikers wel of niet betrokken raken bij een implementatie<br>-Uitspraken over de stakeholders die *vooroplopen* bij de implementatie van de interventie<br>-Uitspraken over de eindgebruikers die *vooroplopen* in de deelname aan een interventie. Bijvoorbeeld omdat ze digitaal vaardig zijn<br><u>Exclusie:</u><br>-Uitspraken die aangeven of een organisatie wel of niet een goed beeld heeft van de behoeften en middelen van de eindgebruikers van de interventie<br>-Uitspraken die aangeven of een nauwkeurig beeld van de behoeften van de eindgebruikers van de interventie al dan niet de implementatie of adoptie van een	• Hoe kijken jullie aan tegen online interventies voor ouderen?

			interventie beïnvloedt _> **Behoeften en middelen van de eindgebruikers van een interventie 2A**	
**5C**	**Uitvoering**	Uitvoeren en volbrengen van de implementatie volgens plan	<u>Inclusie:</u><br>-Uitspraken die laten zien hoe de implementatie wordt uitgevoerd volgens plan	• Geen specifieke vragen in interviewguide
**5D**	**Reflectie & Evaluatie**	-Kwantitatieve en kwalitatieve feedback m.b.t. de voortgang en kwaliteit van de implementatie<br>–Reguliere nabespreking van voortgang en ervaringen m.b.t. de implementatie	<u>Inclusie:</u><br>-Uitspraken van het projectteam dat de implementatie van de interventie uitvoert/aanstuurt m.b.t. de voortgang , de impact en de interpretatie van de uitkomsten van de implementatie van de interventie<br><br><u>Exclusie:</u><br>- Uitspraken over de communicatie over de strategische doelen binnen een organisatie<br>--Uitspraken waarin feedback wordt gegeven over de mate waarin de doelen zijn behaald of niet<br>-Uitspraken die aangeven dat doelen worden aangepast o.b.v. gegeven feedback -> **Doelen en feedback 3D5**<br>-Uitspraken die deelnemers tijdens het interview maken, bijv. m.b.t het succes van de implementatie **-> Kennis en attitude t.a.v. de interventie 4A**	• Wat voor soort informatie zou je nodig hebben om te bepalen of de implementatie van een interventie als een succes te beschouwen is?<br>• Hoe zou je die informatie kunnen verzamelen?<br>• En op welke termijn na de implementatie zou je deze successen graag willen zien?
**6**	**Overig**		-Onder deze code kun je uitspraken onderbrengen die volgens jou niet aan <u>bovenstaande codes toe te wijzen zijn</u>	
